# Nonlinear response of ecosystem respiration to multiple levels of temperature increases

**DOI:** 10.1002/ece3.4658

**Published:** 2019-01-18

**Authors:** Ning Chen, Juntao Zhu, Yangjian Zhang, Yaojie Liu, Junxiang Li, Jiaxing Zu, Ke Huang

**Affiliations:** ^1^ Lhasa Plateau Ecosystem Research Station, Key Laboratory of Ecosystem Network Observation and Modeling, Institute of Geographic Sciences and Natural Resources Research Chinese Academy of Sciences Beijing China; ^2^ University of Chinese Academy of Sciences Beijing China; ^3^ CAS Center for Excellence in Tibetan Plateau Earth Sciences Beijing China; ^4^ College of Resources and Environment University of Chinese Academy of Sciences Beijing China; ^5^ Peking University Shenzhen Graduate School Shenzhen China

**Keywords:** ecosystem respiration, excitatory effect, experimental warming, nonlinear response, Tibetan Plateau

## Abstract

Global warming exerts profound impacts on terrestrial carbon cycles and feedback to climates. Ecosystem respiration (ER) is one of the main components of biosphere CO_2_ fluxes. However, knowledge regarding how ER responds to warming is still lacking. In this study, a manipulative experiment with five simulated temperature increases (+0℃ [Control], +2.1℃ [warming 1, W1], +2.7℃ [warming 2, W2], +3.2℃ [warming 3, W3], +3.9℃ [warming 4, W4]) was conducted to investigate ER responses to warming in an alpine meadow on the Tibetan Plateau. The results showed that ER was suppressed by warming both in dry and wet years. The responses of ER to warming all followed a nonlinear pattern. The nonlinear processes can be divided into three stages, the quick‐response stage (W1), stable stage (W1–W3), and transition stage (W4). Compared with the nonlinear model, the linear model maximally overestimated the response ratios of ER to warming 2.2% and 3.2% in 2015 and 2016, respectively, and maximally underestimated the ratio 7.0% and 2.7%. The annual differences in ER responding to warming were mainly attributed to the distinct seasonal distribution of precipitation. Specially, we found that the abrupt shift response of ER to warming under W4 treatment in 2015, which might be regulated by the excitatory effect of precipitation after long‐term drought in the mid‐growing season. This study highlights the importance of the nonlinearity of warming effects on ER, which should be taken into the global‐C‐cycling models for better predicting future carbon–climate feedbacks.

## 1 INTRODUCTION

Under global warming, the earth's surface temperature has increased 0.76°C since the industrial revolution and is expected to increase to 1.1–3.1°C by the end of this century (Stocker et al., 2013). Global warming affects terrestrial carbon cycles, which can cause positive or negative feedbacks to future climates (Brient & Bony, 2013; Luo, 2007; Melillo et al., 2002). Ecosystem respiration (ER) is one of the largest terrestrial carbon fluxes (Luo, 2007). The model simulation and field observations showed that the annual variation of CO_2_ concentration in atmosphere is closely related to the ER fluctuation (Cox, Betts, Jones, Spall, & Totterdell, 2000; Kato et al., 2004; Luo, 2007; Niu, Sherry, Zhou, & Luo, 2013). Therefore, understanding how ER responds to climatic change is critical for predicting the carbon–climate feedbacks at regional to global scales.

Warming could stimulate ecosystem carbon release across various terrestrial biomes in simulated warming experiments (Niu et al., 2013; Wan, Hui, Wallace, & Luo, 2005). This is largely attributable to that elevated temperature could directly stimulate root and microbial respiration (Niu et al., 2008). However, warming does not necessary result in increasing in ER, because other biotic and abiotic factors could regulate their responses (Wan, Norby, Ledford, & Weltzin, 2007). Water availability may play a predominant role in regulating ER responses to warming, especially in arid and semiarid regions (Xia, Niu, & Wan, 2009). Distinct effects of warming on ER under a soil water gradient were reported in tundra ecosystem (Welker, Fahnestock, Henry, O'Dea, & Chimner, 2004). Lower soil water availability related with warming will exacerbate water limitations, offsetting parts of positive warming effects (Niu et al., 2008). A growing body of evidences demonstrated that climate warming could alter plant community structure and composition (Botkin et al., 2007; Keryn & Mark, 2009). Warming effects on ER vary with plant species (Xia et al., 2009) and functional groups (Niu et al., 2013). Except for these factors, low‐ and high‐level warming induce different changes in soil water availability, water use efficiency (Quan et al., 2018), and community composition (Li, Wang, Yang, Gao, & Liu, 2011) and may lead to distinct ER responses to warming magnitudes. However, warming effects on ER were largely studied in two level warming (control and warming), and consequently reveal simple linear increasing (Niu et al., 2013), linear decreasing (Fu et al., 2013), or no change (Chen et al., 2016; Lin et al., 2011; Xia et al., 2009) of warming effects. To improve our understanding about responses of ER to warming, we need experiments with multiple levels of temperature increases to investigate nonlinear responses (Luo, 2007). To date, related studies for alpine ecosystem distributed in extreme environments are in severe shortage.

**Figure 1:**
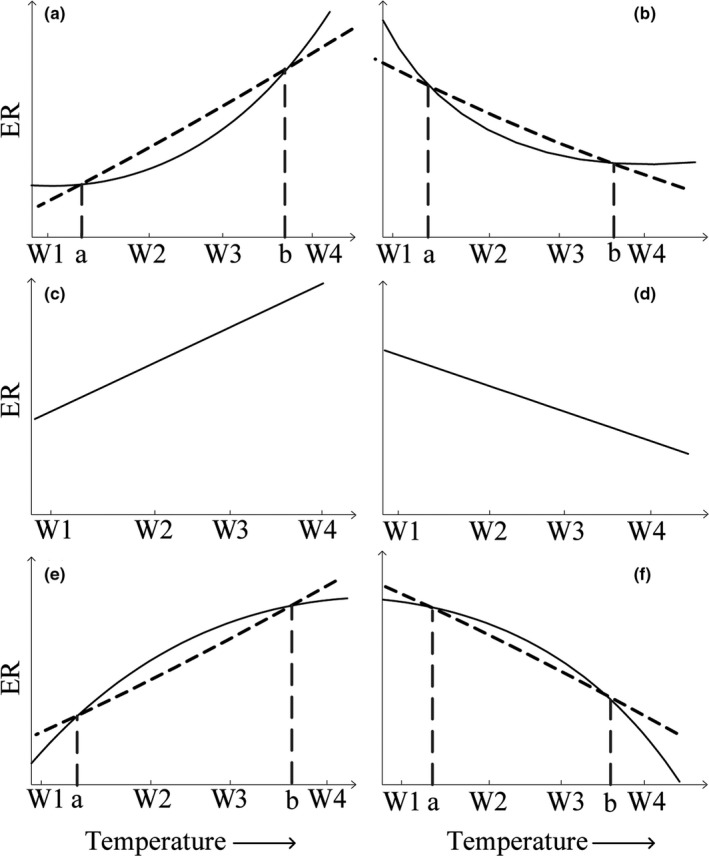
Conceptual diagram of the response of ecosystem respiration (ER) to warming. Dashed line and solid line represent regression equations (solid line: nonlinear regression equations; dashed line: linear regression equations), which evaluate the response of ER to warming

There are six types of possibilities in terms of ER response to a particular temperature range (warming 1 to warming 4 [W1–W4]; Figure 1). When ER nonlinearly increases or decreases with temperature increases, linear models may underestimate ER response to warming under W1‐a and b‐W4 (Figure 1a,b). Warming effects on ER were overestimated by linear models in a–b (Figure 1a,b). If curves are convex, compared with nonlinear models, linear models may overestimate ER response to warming under W1‐a and b‐W4 (Figure 1e,f), and underestimate this effect under a–b (Figure 1e,f). Of course, ER may linearly increase or decrease in response to warming (Figure 1c,d). In summary, responses of ER to warming may vary with warming range. Therefore, nonlinear models may reasonably reveal responses of ER to warming in multiple levels of temperature increases.

There is growing evidence at global, regional, and local scales that interannual precipitation regimes have already become more extreme (Knapp et al., 2008), particularly in arid and semiarid regions. Importantly, transient CO_2_ release ascribable to the “Birch” effect in response to precipitation pulses is a notable property of arid and semiarid ecosystems (Birch, 1958). This indicates carbon fluxes could respond quickly to precipitation events (Huxman et al., 2004). Previous studies have examined priming effects of precipitation pulses on soil respiration after drought in arid or semiarid ecosystems (Austin et al., 2004; Liu, Wan, Su, Hui, & Luo, 2002; Smart & Peñuelas, 2005). The increased soil respiration caused by priming effect contributes 16%–21% of annual total soil respiration (Lee, Nakane, Nakatsubo, Mo, & Koizumi, 2002). It is important to note that “Birch” effect is closely related to soil water condition, showing that precipitation pulses stimulate soil respiration more strongly in drier soil than that in wetter soil (Wang et al., 2010). The above‐mentioned studies all reported priming effects of precipitation pulses on soil respiration, particularly for dry soils. However, knowledge on what role this stimulating effect plays in regulating responses of ER to warming, and how the effects would influence the nonlinear response of ER to warming is even more unclear.

Studies on effects of climate warming on alpine ecosystems have been plentifully conducted on the Tibetan Plateau (Chen et al., 2016; Fu et al., 2013; Lin et al., 2011). However, few studies have examined responses of ER to a warming gradient. The objectives of this study were to address the following: If the nonlinear model could capture the responses of ER to warming, and what processes should be included?

## 2 MATERIALS AND METHODS

### 2.1 Study site

The study was conducted at the Tibet Grassland Ecosystem Research Station (Nagqu station) (31°38.513′N, 92°0.921′E, 4,585 m). The vegetation is typical alpine meadow, dominated by *Kobresia pygmaea*, and accompanied by *Potentilla saundersiana*, *Potentilla cuneata,* and *Stipa purpurea* (Zhu, Zhang, & Jiang, 2017). The long‐term mean annual temperature and precipitation is −1.28℃ and 430 mm (1955–2016), respectively. The growing season normally starts in mid‐May and lasts until mid‐September.

### 2.2 Experimental design

Open‐top chambers (OTCs) were used as passive warming devices based on the International Tundra Experiment design (Marion et al., 1997). The OTCs used in the current study were similar to those in other studies (Chen et al., 2016; Dabros & Fyles, 2010; De Frenne et al., 2010). Warming effects were regulated through changing the heights of OTCs. The treatments in this study include control (C), W1, W2, W3, and W4 (*n* = 3 per treatment). The OTCs were set up in October 2013 and made of 6 mm thick solar transmitting material. They are conical in shape, and are 40 cm (W1), 60 cm (W2), 80 cm (W3), 100 cm (W4) in height, respectively. The top sides of each OTC are 80 cm in order to maintain the same size. The bottom sides are 100 cm (W1), 110 cm (W2), 120 cm (W3), and 130 cm (W4) and cover an area of 2.60 m^2 ^(W1), 3.14 m^2 ^(W2), 3.74 m^2^ (W3), and 4.39 m^2 ^(W4) at the ground, respectively. The 15 plots are separated by a 3.5‐m buffer and arranged following a randomized block design.

### 2.3 Data collection

In October 2014, cylindrical PVC rings (Diameter 9 cm and Height 5 cm) were inserted into soils to a depth of approximately 3 cm and emerge aboveground 2 cm. The ER were measured with an infrared gas analyzer (LI‐6400; LiCor Inc., Lincoln, NE, USA) attached to a respiration chamber. The measurements were implemented from early June to early September, with an interval of approximately 5 days. In each measurement, we firstly obtained stabilized CO_2_S_ml in the natural state, and then set it as the target value. Second, after steady‐state conditions, we set the delta value as 10 ppm. After instrument reduces CO_2_ concentration to below target value of 10 (target value −10) within the chamber, it starts to work, and above target value of 10 (target value +10), it stops to work. This processes cycle three times in each plot. Each measurement was conducted during 9:00 and 12:00 a.m. of sunny days. Totally, 15 times of measurements were accomplished in 2015 and 2016, respectively.

Aboveground biomass was collected by clipping vegetation samples from 0.25 × 0.25 m sections (adjacent to the aluminum frame) at the peak growing season (Aug 22, 2015 and Aug 15, 2016). After clipping, all aboveground plant matter was oven dried at 65℃ for 72 hr before being weighed. Three soil columns with a diameter of 7.0 cm were drilled at depths of 0–10, 10–20, and 20–30 cm.

A 1 × 1 m frame with 100 equally distributed grids (0.1 × 0.1 m) was placed above the vegetation canopy to measure vegetation coverage (1 × 1 m). Grids with plants appearing over 1/2 of the grid were marked as 1, otherwise marked as 0. The total number of grids within the frame is the actual coverage value. The cover was mainly measured in mid‐growth season and late growth season and was accomplished 2–3 times in both growing seasons.

Air temperature and moisture at 10 cm aboveground were measured using the Vaisala HMP155A sensor (Vaisala, Helsinki, Finland). Soil temperature and moisture at 5 cm belowground were measured at the centers of each plot using Campbell CS655 sensors (Campbell Scientific, Logan, UT, USA). Measurements of air temperature, soil temperature, and soil moisture were taken with 30‐min intervals, and averages of the two measurements were stored as the hourly averages, and averages of the 48 measurements were the day averages. In each warming treatment (three plots), we installed air and soil sensors (soil temperature and soil moisture) in two of them, and used average of the two measurements (Zhu et al. 2017).

### 2.4 Statistical analysis

Repeated‐measures ANOVA (RMANOVA) were used to examine warming effects on ER over the growing seasons in 2015 and 2016. The between‐subject effects were treated as warming effect and the within‐subject effects were time‐of‐season. To analyze the seasonal variations of ER response, the whole growing season was divided into two stages, early‐growing season and late growing season. Then, one‐way ANOVA was applied to analyze the treatment difference for ER at two stages and the whole growing season in 2015 and 2016. The curve estimation was employed to analyze the relationship between ER and soil temperature and soil moisture. All statistical analyses were conducted with SPSS software (SPSS 20.0 for windows).

To examine the nonlinear responses, we calculated the response ratios of treatment (W1, W 2, W3, W4) to control (no temperature increase) based on the mean values of the 15 measurements during the 2015 and 2016 growing seasons. The response of ER to temperature increase was fit with two types of models: linear and nonlinear model. The linear and nonlinear function in R 3.1.0 was used to estimate the nonlinear and linear model coefficients, respectively. The fitness of the models was compared based on coefficient of determination (*R* ^2^), Akaike's information criterion (AIC), and Bayesian information criterion (BIC). The larger *R* ^2^ value and the smaller AIC and BIC values in the linear and nonlinear model indicate a better fit (Wang et al., 2014). The R 3.1.0 was used in model fitting and estimation. The Sequential Mann–Kendall (SQMK; Sayemuzzaman & Jha, 2014) test and one‐way ANOVA were used to estimate the position of the breakpoint.

## 3 RESULTS

### 3.1 Response of microclimate to warming

**Figure 2:**
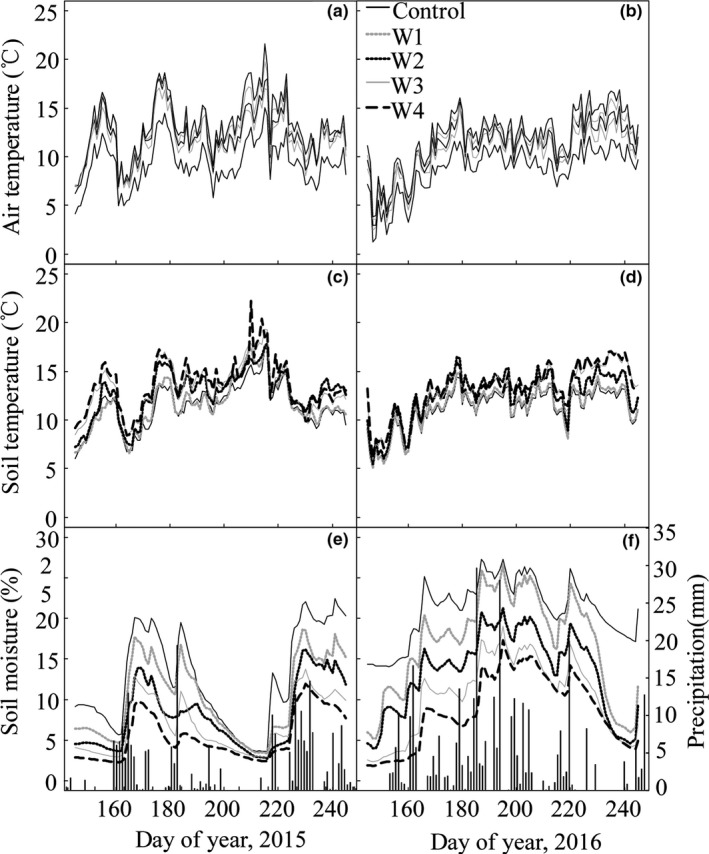
Air temperature (°C, a: 2015; b: 2016), soil temperature at 5 cm depth (°C, c: 2015; d: 2016), soil moisture (%, e: 2015; f: 2016) at 5 cm depth and daily precipitation (mm, bars) during the growing season under different warming treatments

Mean seasonal air temperature was 2.4℃, 2.9℃, 3.4℃, 3.9℃ in 2015 (Figure 2a) and 1.7℃, 2.2℃, 2.7℃ and 3.4℃ in 2016 (Figure 2b) higher in warmed plots (W1, W2, W3, and W4) than control plots, respectively. Mean seasonal soil temperature was 0.4℃, 1.5℃, 1.9℃, 2.4℃ in 2015 (Figure 2c), 0.4℃, 1.6℃, 2.1℃, 2.5℃ in 2016 (Figure 2d) higher in warmed plots (W1, W2, W3, and W4) than control plots, respectively. Soil moisture was on average lowered by 2.5%, 4.8%, 5.9%, 7.4% in 2015 (Figure 2e), 3.8%, 7.6%, 10.6%, 12.4% in 2016 (Figure 2f) under warming treatments (W1, W2, W3, and W4), respectively.

Both years were characterized by contrasting precipitation patterns. Total precipitation in 2015 (300.8 mm) was 30.7% lower than the long‐term mean annual precipitation (MAP; 433.3 mm). Total precipitation in 2016 (551.8 mm) was 27.2% higher than the MAP. Precipitation also exhibited strong seasonal patterns in 3 years. In spring and summer, precipitations were less than the MAP (Figure 2e). Particularly, in July of 2015, precipitation was 63.2% lower than the MAP (Figure 2e). In growing season of 2016, precipitation exhibited a single peak patterns, and the summer total was 39.1% higher than that of the MAP (Figure 2f).

### 3.2 Response of ER to warming during both growing seasons

**Figure 3:**
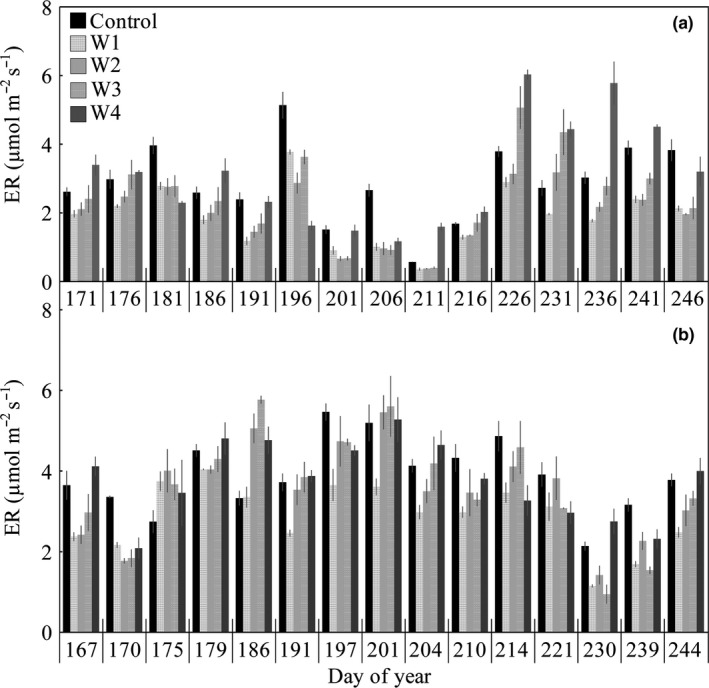
Seasonal dynamics (mean ± 1 *SE*) of ecosystem respiration (ER) in 2015 (a) and 2016 (b)

Mean seasonal ER decreased by 34.4% (*p* < 0.1; W1), 31.1% (*p* < 0.1; W2), 14.6% (*p* > 0.1; W3) but increased by 6.7% (*p* > 0.1; W4) under warming treatments in 2015, respectively (Figure 3a). Under warming treatments, ER was decreased by approximately 25.8% (*p* < 0.1; W1), 9.7% (*p* > 0.1; W2), 9.9% (*p* > 0.1; W3), and 2.8% (*p* > 0.1; W4) compared with control subplots in 2016, respectively (Figure 3b).

**Figure 4:**
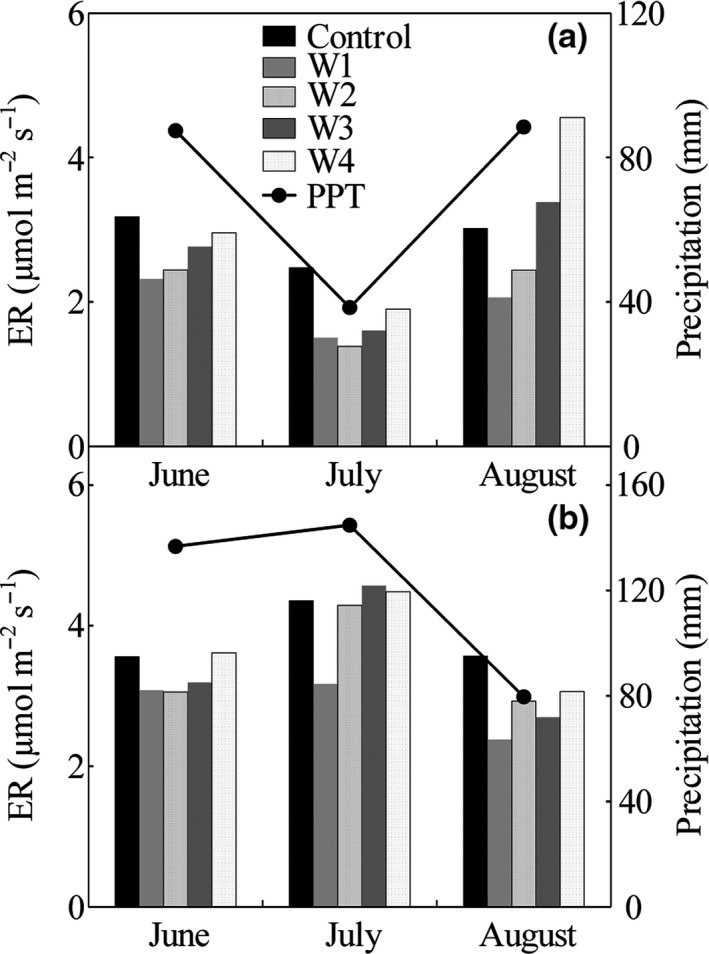
Monthly precipitation (lines) and ecosystem respiration (ER) (bars) in 2015 and 2016

Mean growing season ER in wet growing season (2016) was 34.3% (*p* < 0.1; control), 52.1% (*p* < 0.1; W1), 67.8% (*p* < 0.1; W2), 45.0% (*p* > 0.1; W3), and 22.4% (*p* < 0.1; W4) greater than dry growing season (2015), respectively (Figure 3). The temporal dynamics of ER were in accord with seasonal patterns of precipitation in both years, which were lower in summer and higher in autumn of 2015, and were higher in summer and lower in spring and autumn of 2016 (Figure 3). We further found that monthly mean ER between growing seasons coincided with monthly precipitation (Figure 4). These results may suggest that controls of precipitation pattern on seasonal variations in ER.

### 3.3 Model fitting of the response of ER to air temperature changes

**Figure 5:**
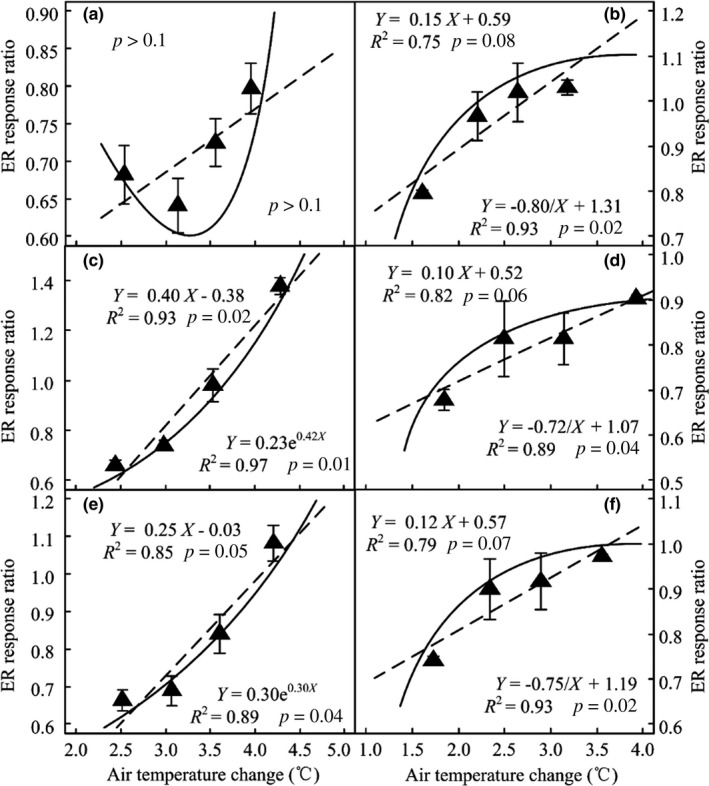
Relationships between the response ratios of ecosystem respiration (ER) and air temperature changes at early season (a, b), late growing season (c, d), and the whole growing season (e, f) in 2015 (left) and 2016 (right). Dashed line and solid line represent regression equations (dashed line: linear regression equations; solid line: nonlinear regression equations) between the response ratios of ER to warming and air temperature changes

*R* ^2^, AIC, and BIC values showed nonlinear models performed better than linear models for ER response to warming in 2015 and 2016, except for the early‐growing season in 2015 (Figure 5a; Table 1). Compared with the nonlinear model, the linear model underestimated the response ratios of ER to warming 7.0% and 4.1% at 2.4–2.8°C and 3.8–4.3°C, respectively, during late growing season of 2015 (Figure 5c; Table 2). However, the nonlinear model overestimated the response ratio 2.2% at 2.8–3.8°C warming (Figure 5c; Table 2). For the whole growing season of 2015, the linear model overestimated the response ratios by 1.1% at 3.0–3.9°C warming and underestimated by 3.7% and 1.1% at 2.5–3.0°C and 3.9–4.2°C warming, respectively (Figure 5e; Table 2).

**Table 1:** Coefficient of determination (Adjusted *R* ^2^), Akaike's information criterion (AIC) and Bayesian information criterion (BIC) from two different models predicting the effects of warming on ecosystem respiration in 2015 and 2016

	Growing season	Early‐growing season	Late growing season
2015			
*R* ^2 ^(linear)	0.85	–	0.93
*R* ^2 ^(nonlinear)	0.89	–	0.97
AIC (linear)	−6.40	–	−5.14
AIC (nonlinear)	−8.66	–	−10.48
BIC (linear)	−8.24	–	−6.98
BIC (nonlinear)	−10.50	–	−12.32
2016			
*R* ^2 ^(linear)	0.79	0.75	0.82
*R* ^2 ^(nonlinear)	0.93	0.93	0.89
AIC (linear)	−10.15	−8.86	−11.19
AIC (nonlinear)	−14.72	−14.00	−13.14
BIC (linear)	−11.99	−10.70	−13.03
BIC (nonlinear)	−16.56	−15.84	−14.98

Note

– represents the models do not fit success.

**Table 2:** Comparison between the linear and nonlinear response ratios of ecosystem respiration to warming in 2015 and 2016

	Underestimate (℃)	Underestimate (%)	Overestimate (℃)	Overestimate (%)
2015				
E‐S	–	–	–	–
L‐S	2.4–2.8	7.0	2.8–3.8	2.2
	3.8–4.3	4.1		
S	2.5–3.0	3.7	3.0–3.9	1.1
	3.9–4.2	1.1		
2016				
E‐S	1.8–2.9	2.2	1.6–1.8	3.2
			2.9–3.2	1.2
L‐S	2.0–3.6	2.7	1.8–2.0	2.7
			3.6–3.9	1.6
S	1.9–3.3	2.5	1.7–1.9	2.8
			3.3–3.8	3.0

Note

Compared to nonlinear models, the range of linear models underestimate or overestimate ecosystem respiration was displayed by underestimate (℃) and overestimate (℃), respectively, and the maximum overestimate or underestimate are represented overestimate (%) and underestimate (%), respectively.

E‐S: early season; L‐S: late season; S: the whole of season.

Compared with nonlinear model, the linear model overestimated the response ratios of ER by 3.2% and 1.2%, respectively, at 1.6–1.8°C and 2.9–3.2°C warming, and underestimated the response ratios of ER by 2.2% at 1.8–2.9°C warming during early‐growing season of 2016 (Figure 5b; Table 2). Compared with nonlinear model at 1.8–2.0°C and 3.6–3.9°C warming, the linear model overestimated the response ratios of ER by 2.7% and 1.6%, respectively, at late growing season in 2016 and underestimated the response ratios of ER by 2.7% at 2.0–3.6°C warming (Figure 5d; Table 2). During the growing season of 2016, the linear model overestimated the response ratios of ER by 2.8% and 3.0% at 1.7–1.9°C and 3.3–3.8°C warming, respectively, and underestimated by 2.5% at 1.9–3.3°C warming (Figure 5f; Table 2).

### 3.4 Nonlinear responses of ER to warming

**Figure 6:**
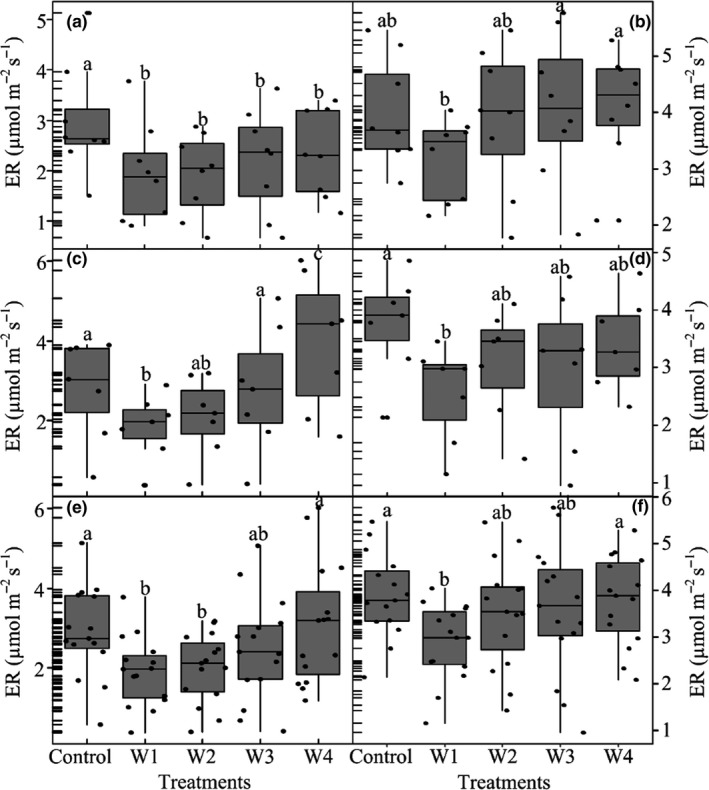
Seasonal means of ecosystem respiration (ER) at early season (a, b), late growing season (c, d), and the whole growing season (e, f) under warming treatments in 2015 (left) and 2016 (right). Different letters in figures indicate significant difference (*p* < 0.1)

Nonlinear response processes of ER to warming are composed of sensitive response, stable response, and transition response. ER response to warming was more sensitive to low temperature increase (W1). ER under W1 treatments was 34.4% and 25.8% (*p* < 0.1) lower than under control treatments, respectively, in 2015 and 2016 (Figure 6e,f). ER exhibited a stable response to warming, which shows that warming exerted no significant effects on ER from W1 to W3 in 2015 and from W1 to W4 in 2016 (Figure 6e,f). This stable response was also found at early‐ and late growing season in 2015 and 2016, respectively (Figure 6a–d).

**Figure 7:**
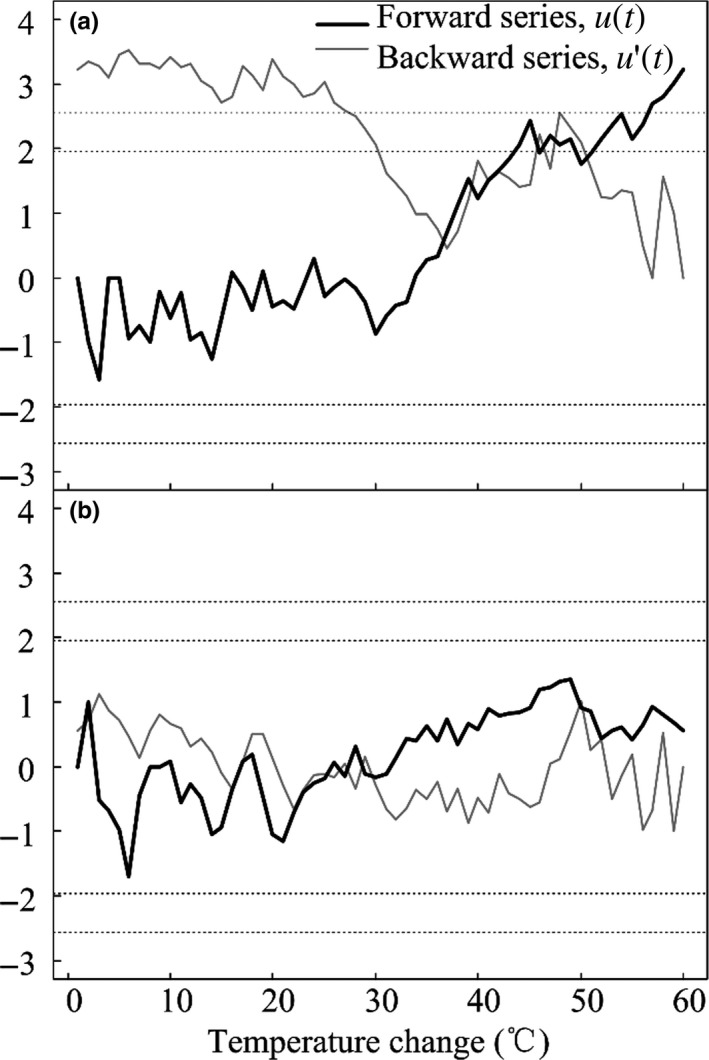
Sequential Mann–Kendall (SQMK) test to predict the abrupt shift temperature change data series of the stations of 99% and 95% confidence level (horizontal dotted line) MK statistics for 2015 (a) and 2016 (b) growing seasons

At late growing season of 2015, W4 significantly stimulated ER by 41.3% (control), 115.4% (W1), 89.6% (W2) and 41.8% (W3), respectively (Figure 6c). Compared with ER mean in stable stages (W1–W3), ER significantly increased in W4 in the whole growing season and late growing season of 2015 (Table 3; *p* < 0.1). This indicated that transition response was observed under W4. However, such a transition was not identified in 2016 (Table 3, *p* > 0.1). The SQMK test showed that there was an abrupt shift in ER with temperature changes. The abrupt positive trend shift of ER occurred around 4.2℃, and reached significant around 4.7℃ in 2015 (Figure 7a), whereas no significant abrupt shift was identified in 2016 (Figure 7b). Temperature increases greater than 4.2 or 4.7℃ were concentrated in the W4 treatment, suggesting the abrupt positive trend shift of ER in W4.

**Table 3:** *F* values for one‐way ANOVA of variance for ecosystem respiration under W4 treatment and during the stable stage in 2015 and 2016

	Growing season	Late growing season	Early‐growing season
2015	4.27**	36.81***	–
2016	2.82	–	0.10

Note

In this table, we only list the situations that compared W1, W2 or W3 (at least one of the three), W4 treatments significantly increased ER. Because only in this context, W4 may exist to break the stable state that composition of the W1, W2, W3 treatments (between the three were not significant).

–: it represents not have this situation.

Significance:

* *p* < 0.10.

** *p* < 0.05.

*** *p* < 0.01.

### 3.5 Impacts of abiotic factors on ER

**Figure 8:**
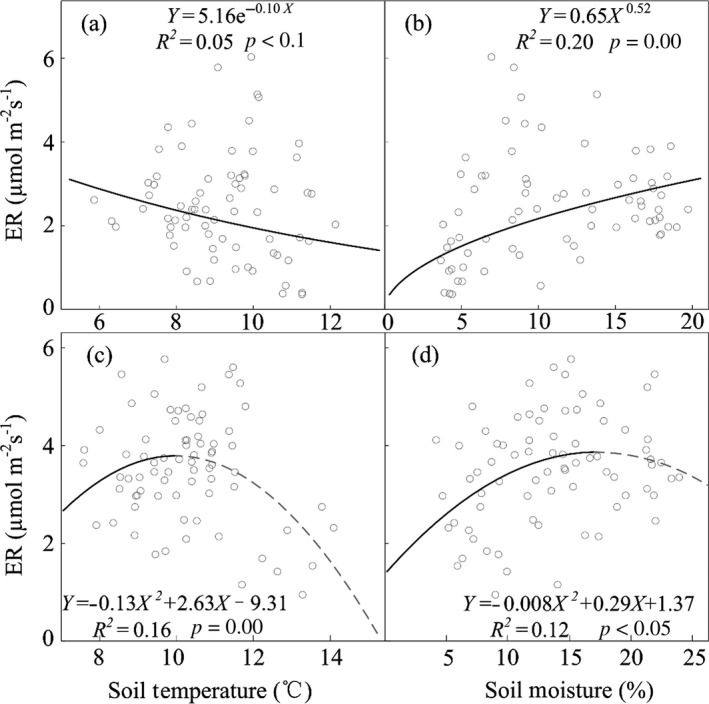
Relationships between ecosystem respiration (ER) and soil temperature (a, c), soil moisture (b, d) in 2015 (above) and 2016 (below). Dots represent the mean value under different warming treatments

The exponential model indicated that ER negatively correlated with soil temperature, which explained approximately 5% of variations in ER over 2015 growing season (Figure 8a). Similar to 2015, the quadratic model revealed that ER also negatively correlated with soil temperature, and 16% variations in ER were explained over 2016 growing season (Figure 8c). The ER was positively correlated with soil temperature when it was lower than 10.12℃, but negatively correlated with soil temperature when it was higher than 10.12℃ (Figure 8c). The power model and quadratic model indicated that ER positively correlated with mean soil moisture in both years (Figure 8b,d). What is more, ER positively correlated with soil moisture when it was lower than 18.13%, but negatively with soil moisture when it was higher than 18.13% in 2016 (Figure 8d).

## 4 DISCUSSION

### 4.1 Negative nonlinear response of ER to warming

**Figure 9:**
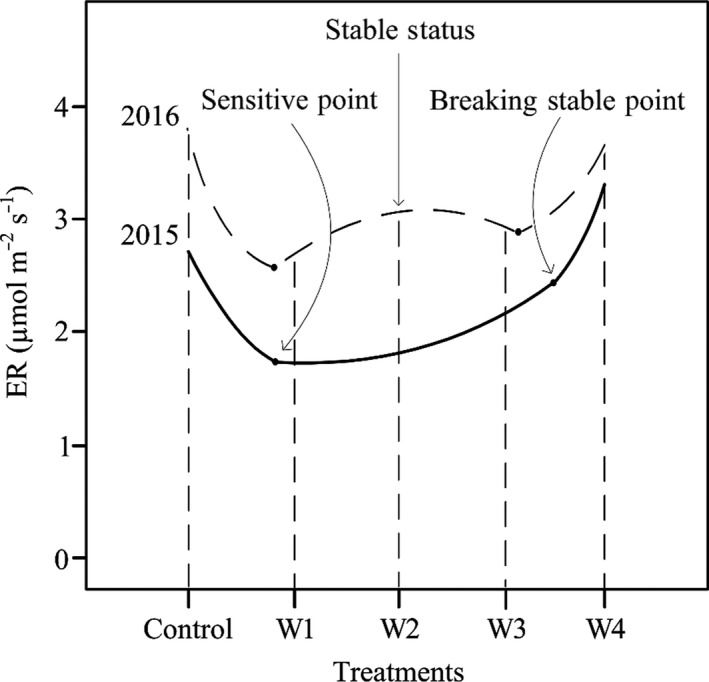
Conceptual diagram of the nonlinear processes of ecosystem respiration (ER) to warming, including sensitive point, stable status and transition point. Solid and dashed lines represent 2015 and 2016, respectively

This study explicitly revealed that response of ER to warming all followed a nonlinear pattern, a feature that previous studies failed to capture (Chen et al., 2016; Fu et al., 2013; Grogan & Iii, 2000; Hobbie & Iii, 1998; Lin et al., 2011; Niu et al., 2013; Welker et al., 2004; Xia et al., 2009). More importantly, the nonlinear responses of ER to warming were made up of three stages, including sensitive response, stable state response, and transition response (Figure 9).

Warming can exert negative effects on ER primarily through limiting soil water availability, especially in arid and semiarid regions (Xia et al., 2009). Lower soil water availability would restrict root, microbial activities, translating into reduced ER (Niu et al., 2008). These passive effects have reported in same study area (Zhu et al., 2017). Resent study reveals water availability more than temperature drives carbon fluxes of alpine meadow (Zhu et al.,^2017^). In this study, positively linear dependence of ER upon soil moisture further supports these findings.

Plant growth effects on carbon fluxes are modulated by soil water availability in growing season (Liu, Cieraad, Li, & Ma, 2016). For the alpine meadow ecosystem, *K. pygmaea*, as a dominant species, is shallow‐rooted, and mostly utilize shallow soil water (Dorji et al., 2013). More importantly, it is a drought vulnerable species (Li, Wang, Yang, Gao, Liu, & Liu, 2011), and warming further exacerbate the vulnerability. Thus, warming significantly decreases *K. pygmaea* coverage (Supporting Information Figure S1). Decreased plant cover could decrease canopy cover and increase bare soil evaporation, and consequently decrease aboveground plant respiration (Verburg et al., 2004; Supporting Information Figure S2). In addition, *K. pygmaea* belongs to dense bush fibrous root perennial plant, which could form a huge underground biomass (Liu, Sun, Zhang, Pu, & Xu, 2008). The negative effects of warming on *K. pygmaea* could decrease underground biomass (Chen et al., 2018), translating into reduced below‐ground plant respiration. For alpine ecosystems, ER variations are controlled by plant respiration (Chen et al., 2016). In addition, nonlinear relationships between abiotic factors and ER may cause the nonlinear response of ER to warming in this study.

### 4.2 Sensitive response to warming under low temperature increase

The ER was more sensitive to warming under low temperature increase (W1) for the alpine ecosystem (Supporting Information Figure S3). The temperature sensitivity of ER is mainly related to temperature range (Lin et al., 2011; Tjoelker, Oleksyn, & Reich, 2001) and soil moisture (Flanagan & Johnson, 2005; Reichstein et al., 2002; Wen et al., 2006). The temperature sensitivity of ER was significantly affected by soil temperature, and weakened with increased temperature (Supporting Information Figure S4). This result is in accord with previous studies (Bekku, Nakatsubo, Kume, Adachi, & Koizumi, 2003; Lin et al., 2011; Zheng et al., 2009; Zhou, Wan, & Luo, 2007). However, soil moisture and vegetation coverage stimulated the temperature sensitivity of ER. The quick changes in soil temperature, moisture, and vegetation coverage may lead to higher sensitivity of ER to warming under low temperature increase (W1). In turn, the greater sensitivity of ER to warming suggests that the alpine ecosystem, including vegetation and soil system, may experience large changes under future climate change.

### 4.3 Stable and transition response to warming under high temperature increase

Our results demonstrated that ER exhibited stable and transition response to warming as temperature increases in 2015. But the transition response was not found in 2016. These contrasting responses may be related to the distinct seasonal precipitation distribution during the growing season. Previous studies have shown that the fluctuation of ER is regulated by the seasonal distribution of precipitation (Marcolla et al., 2011; Nijp et al., 2014; Ryan et al., 2015). For example, with decreased precipitation, the declined ER was coherent with the lower precipitation and soil moisture in 2015 in this study (Figure 3).

At the early‐growing season of 2015, soil moisture continued to decline due to reduced precipitation. The stimulating effects of warming on soil respiration were offset by the negative effect of water deficiency (Bontti et al., 2009). Soil respiration accounts for 85% of ER in this study (data not shown). Consequently, warming had no significant effects on CO_2_ emission of ecosystem (Bontti et al., 2009), and ER exhibited a flat response to warming. In contrast, adequate precipitation in growing season of 2016 resulted in sufficient soil moisture. Further increase in precipitation did not continue to rise in respiration (Liu, Zhang, Zhen‐Zhu, Zhou, & Hou, 2012), even decreased it (Cavelier & Penuela, 1990). ER negatively correlated with soil moisture when soil moisture was higher than 18.13% in this study. The standardized major axis estimation regression showed that no significant change in regression slopes between ER and soil moisture were found from W2 to W4 (Supporting Information Table S1). This could further supply an additional explanation for the stable response of ER to warming in 2016.

Warming can cause profound impacts on ER by decreasing soil moisture and increasing temperature (Frey, Drijber, Smith, & Melillo, 2008; Lin et al., 2011; Xu, Sherry, Niu, Zhou, & Luo, 2012), which would restrict plant photosynthesis, root growth, and respiration (Domec & Gartner, 2003) and affect microbial activities, ultimately, reducing the amount of CO_2_ (Liu, Zhang, & Wan, 2009). Precipitation can improve soil moisture conditions and rapidly stimulate soil respiration, and the effect usually lasts 2–6 days (Clein & Schimel, 1994; Franzleubbers, Stuedemann, Schomberg, & Wilkinson, 2000; Xu, Baldocchi, & Tang, 2004). Even small amounts of precipitation can strongly accelerate soil respiration (Birch, 1958; Franzleubbers et al., 2000), and moderate levels of precipitation and duration have a stronger stimulating effect on soil respiration (Huxman et al., 2004). In addition, the lower soil moisture content was, the stronger the excitatory effect was (Liu et al., 2002; Shi et al., 2006). Therefore, the increase in soil respiration triggered by precipitation pulse is proportional to drought time and inversely proportional to soil respiration rate before precipitation (Xu et al., 2004).

In 2015, total precipitation was only 34.8 mm between July 4 and August 10 (DOY: 185–222), leading to severely decreased soil water. Compared with ER in August 12th (DOY: 224), it was promptly elevated in August 16th (DOY: 228) by 2.26 times, 2.23 times, 2.33 times, 2.95 times, and 2.97 times in control, W1, W2, W3, and W4, respectively. This phenomenon was largely attributable to precipitation pulse in this stage (DOY: 224–227; 36.3 mm). More importantly, precipitation pulse continues to stimulate driest soil conditions in late growing season of 2015. As a result, ER under W4 was greater than under other warming treatments after August 16th (DOY: 226). Results showed that ER in W4 was 114.88% (W1), 86.81% (W2), and 38.17% (W3) higher than under other warming treatments, respectively (*p* < 0.1) in late growing season (DOY: 228–248). Therefore, our results highlight that the priming effect of precipitation may result in the transition response of ER to warming under high temperature increase in 2015.

## 5 CONCLUSIONS

By conducting a warming experiment on an alpine meadow over two growing seasons, this study showed that ER displayed a nonlinear pattern with temperature increases. Further, the nonlinear processes could be divided into three stages during the dry growing season. First, ER was more sensitive to low temperature increase, which may be attributed to the quick changes in soil conditions and vegetation coverage. Second, ER displayed a flat response to warming due to the combined effect of biotic and abiotic factors. Finally, precipitation at late growing season could rapidly stimulate soil respiration. The flat state was broken by the excitatory effect of precipitation under high temperature increase. This study demonstrated the nonlinearity is likely a general feature for ER in response to warming, and these nonlinear processes and regimes should be taken into the global‐C‐cycling models for better predicting future carbon‐climate feed backs.

## CONFLICT OF INTEREST

The authors declare no conflict of interest

## AUTHOR CONTRIBUTIONS

Ning Chen, Yangjian Zhang, and Juntao Zhu conceived and designed the study. Ning Chen, Junxiang Li, and Yaojie Liu contributed field measurements. Ning Chen, Ke Huang, and Jiaxing Zu contributed the statistical analysis. Ning Chen and Yangjian Zhang wrote the paper. All authors contributed to the writing the manuscript.

## Data Availability

I agree to deposit the main data of the article in Dryad. https://doi.org/10.5061/dryad.610p0s4.
