# Supplementary material for: Nonlinear response of ecosystem respiration to multiple levels of temperature increases
Source: Ecol Evol. 2019 Jan 18;9(3):925–37. doi: 10.1002/ece3.4658 (PMC6374685; doi:10.1002/ece3.4658)
Supplement: Supplementary file 1 [file ECE3-9-925-s001.docx]

**Nonlinear response of ecosystem respiration to multiple levels of temperature increases**

Ning Chen^1,2^, Juntao Zhu^1*^, Yangjian Zhang^1, 3, 4^, Yaojie Liu^1,2*^, Junxiang Li^1, 5^, Jiaxing Zu^1,2^, Ke huang^1,2^

^1^Lhasa Plateau Ecosystem Research Station, Key Laboratory of Ecosystem Network Observation and Modeling, Institute of Geographic Sciences and Natural Resources Research, Chinese Academy of Sciences, Beijing, 100101, China, ^2^University of Chinese Academy of Sciences, Beijing, 100190, China, ^3^CAS Center for Excellence in Tibetan Plateau Earth Sciences, Beijing 100101, China, ^4^College of Resources and Environment, University of Chinese Academy of Sciences, Beijing, 100190, China, ^5^Peking University Shenzhen Graduate School, Shenzhen 518055, China

**FIGURE S1** The response of *K. pygmaea* coverage to warming in 2015 (black bars) and 2016 (grey bars). Different letters in insets indicate significant difference (*p* < 0.1).

**FIGURE S2** The relationship between ER and *K. pygmaea* coverage.

**FIGURE S3** The temperature sensitivity (TS) of ER (changes in ER per ℃ increase of soil temperature) in 2015 (black bars) and 2016 (grey bars). Different letters in insets indicate significant difference (*p* < 0.1).

**FIGURE S4** Relationships between temperature sensitivity of ecosystem respiration and soil temperature (a), soil moisture (b), vegetation coverage (c), respectively, in 2015 (hollow triangles and dotted lines) and 2016 (solid triangles and solid lines).

**Table S1** Summary of standardized major axis estimation regression parameters for the relationships between carbon fluxes and biotic and abiotic factors in different warming treatments in three years.

|  | *R*^2^ | *P* | Slope |
| --- | --- | --- | --- |
| Control | 0.74 | 0.00 | 0.16 **a** |
| W1 | 0.85 | 0.00 | 0.17 **a** |
| W2 | 0.60 | 0.01 | 0.26 **ab** |
| W3 | 0.34 | 0.10 | 0.43 **b** |
| W4 | 0.23 | 0.20 | 0.34 **b** |

Different letters in table indicate significant difference in slopes (*p* < 0.1).
