# Supplementary figures and images for: Nonlinear response of ecosystem respiration to multiple levels of temperature increases
Source: Ecol Evol. 2019 Jan 18;9(3):925–37. doi: 10.1002/ece3.4658 (PMC6374685; doi:10.1002/ece3.4658)

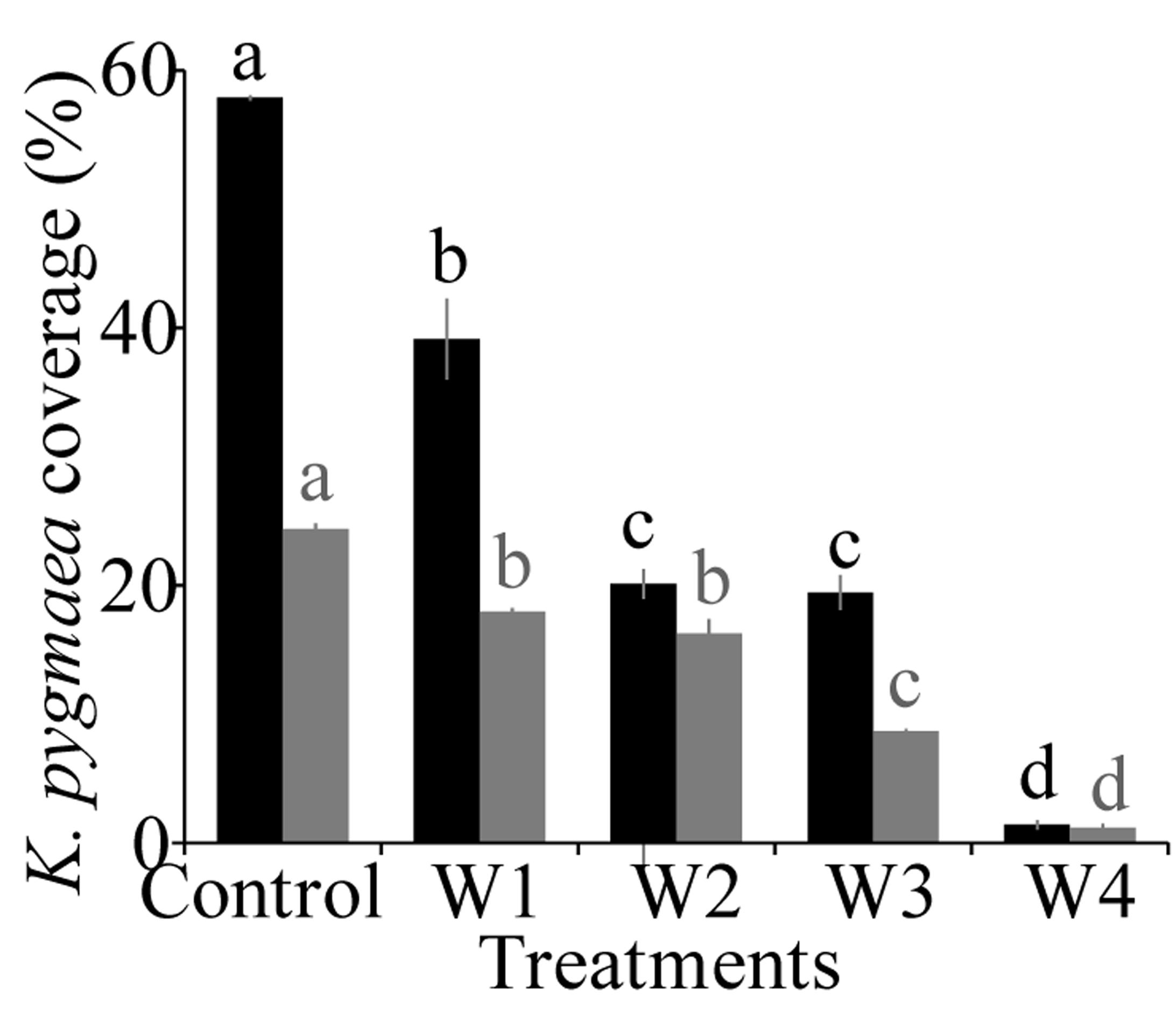

Supplement: Supplementary file 2 [file ECE3-9-925-s002.tif]

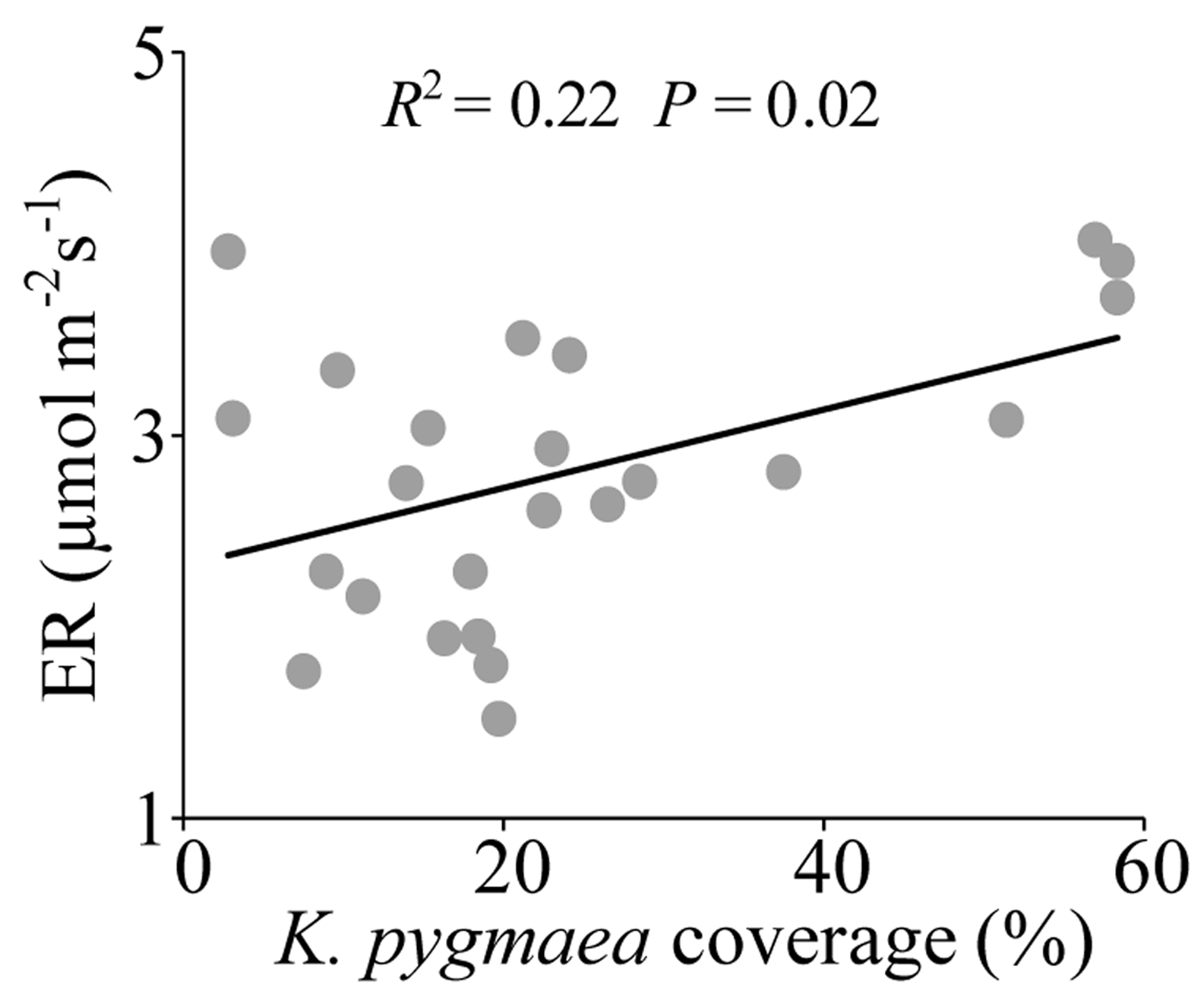

Supplement: Supplementary file 3 [file ECE3-9-925-s003.tif]

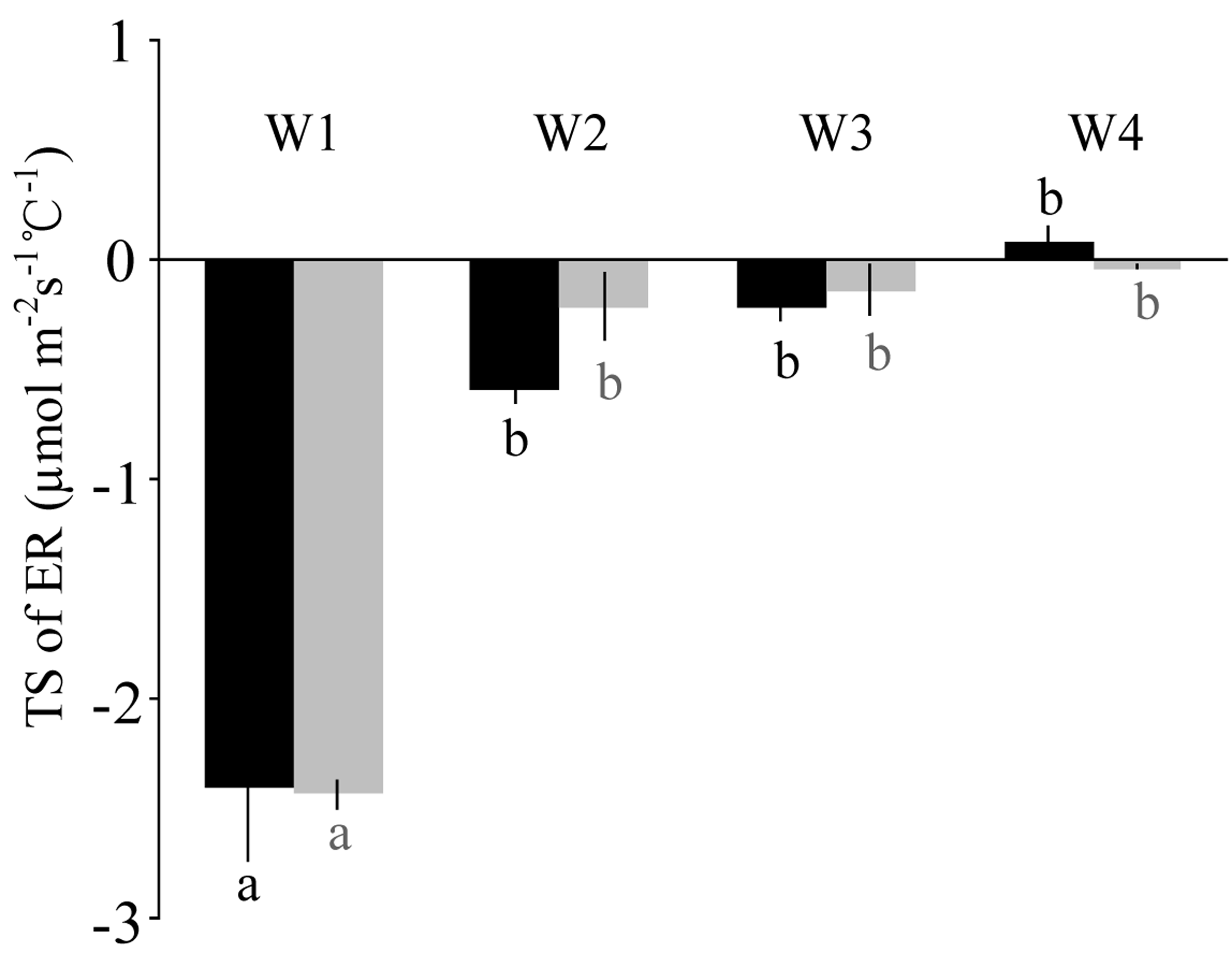

Supplement: Supplementary file 4 [file ECE3-9-925-s004.tif]

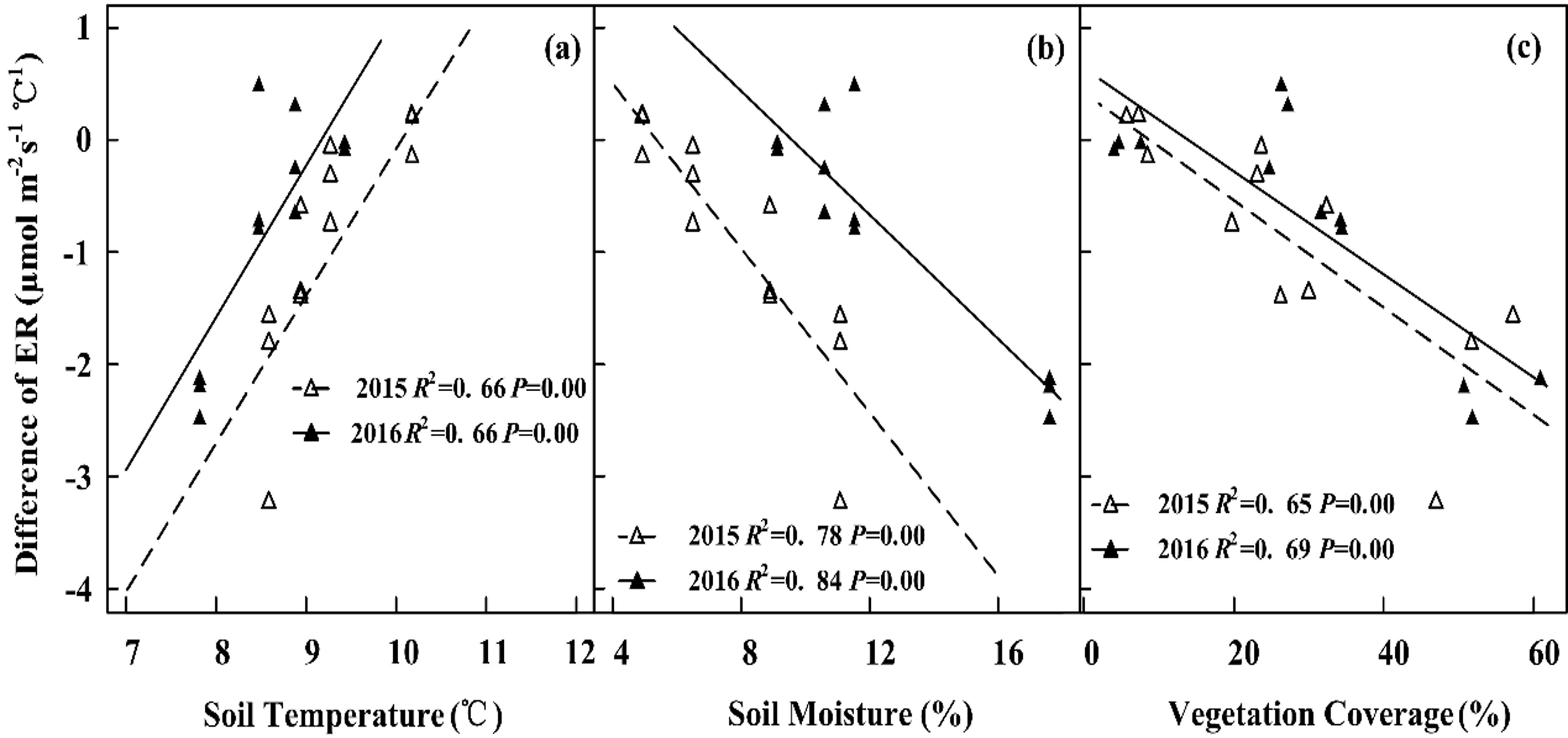

Supplement: Supplementary file 5 [file ECE3-9-925-s005.tif]
